# Micro-Fragmentation as an Effective and Applied Tool to Restore Remote Reefs in the Eastern Tropical Pacific

**DOI:** 10.3390/ijerph17186574

**Published:** 2020-09-09

**Authors:** J. J. Adolfo Tortolero-Langarica, Alma P. Rodríguez-Troncoso, Amílcar L. Cupul-Magaña, Baruch Rinkevich

**Affiliations:** 1 Tecnológico Nacional de México/IT Bahía de Banderas, Crucero a Punta de Mita S/N, Bahía de Banderas, C.P., Nayarit 63734, Mexico; 2 Laboratorio de Ecología Marina, Centro de Investigaciones Costeras, Centro Universitario de la Costa, Universidad de Guadalajara. Av. Universidad No. 203, Puerto Vallarta, C.P., Jalisco 48280, Mexico; pao.rodriguezt@gmail.com (A.P.R.-T.); amilcar.cupul@gmail.com (A.L.C.-M.); 3 Israel Oceanography and Limnological Research, National Institute of Oceanography, Tel Shikmona, P.O. Box 8030, Haifa 31080, Israel

**Keywords:** climate change, coral restoration, nubbins, coral growth, calcification rates, *Pavona clavus*, *Pocillopora*, marginal reef, Mexican Pacific

## Abstract

Coral reef ecosystems are continuously degraded by anthropogenic and climate change drivers, causing a widespread decline in reef biodiversity and associated goods and services. In response, active restoration methodologies and practices have been developed globally to compensate for losses due to reef degradation. Yet, most activities employ the gardening concept that uses coral nurseries, and are centered in easily-accessible reefs, with existing infrastructure, and impractical for coral reefs in remote locations. Here we evaluate the effectiveness of direct outplanting of coral micro-fragments (*Pavona clavus* and *Pocillopora* spp.) as a novel approach to restore remote reefs in the Islas Marías archipelago in the Eastern Tropical Pacific. Coral growth (height-width-tissue cover), survival percentage, extension rates (cm year^−1^), skeletal density (g cm^−3^) and calcification rates (g cm^−2^ year^−1^) were assessed over 13 months of restoration. In spite of detrimental effects of Hurricane Willa, transplants showed a greater-than-twofold increase in all growth metrics, with ~58–61% survival rate and fast self-attachment (within ~3.9 months) for studied species, with Pocilloporids exhibiting higher extension, skeletal density, and calcification rates than *Pavona*. While comprehensive long-term studies are required, direct transplantation methodologies of coral micro-fragments are emerging as time-effective and affordable restoration tools to mitigate anthropogenic and climate change impacts in remote and marginal reefs.

## 1. Introduction

Worldwide distributed coral reef ecosystems host >25% of marine life, sustain important biogeochemical and ecological functionality [1,2], and uphold goods and services for human wellbeing [3,4,5], yet are continuously impacted by the cumulative effects of anthropogenic stressors and climate change drivers, causing a widespread degradation of these valuable coral reef communities [6,7,8]. Several coral reef ecosystems revealed some resistance to the inflicted impacts, with natural recovery processes with slow recovery trajectories, yet, all providing some time for natural acclimatization to occur [9,10,11,12,13,14,15,16]. In response, the notion of active coral reef restoration as a key strategy addressing the cumulative impacts of anthropogenic and climate change has been put forward, and new coral restoration approaches have been developed globally in the last decades [15,17,18,19,20,21,22,23,24,25]. As it becomes questionable whether corals would be able to acclimatize quickly enough [8,26], the emerged active reef restoration measures have acquired further consideration as effective tools for facilitating the rehabilitation of the globally impacted coral reef ecosystems [27,28,29,30,31,32], and to save time, since adaptation seems to occur over slow evolutionary timescales [33,34]. At this point, many of the active reef restoration measures are using ecological engineering applications, gardening and farming concepts, assisted migration/colonization, assisted genetics/evolution, assisted microbiome, epigenetics, and chimerism (reviewed in [33,35]). Although some progress has been made in the last two decades, coral reef restoration is far from being a mature discipline, as many approaches present a local applicability or were not explored in a wide range of coral reefs [15,36]. Hence, the continuous assessment of restoration tools and the development of cost- and time-effective protocols are required to mitigate, or compensate for, rapid coral reef degradation [23,25,33].

The Eastern Tropical Pacific (ETP) coral reef communities are declining, experiencing anthropogenic and climate change impacts, including several major coral bleaching and mortality events, resulting from the El Niño–Southern Oscillation (ENSO) phenomena. Live coral cover has been reduced from 33% to 7% with >90% local coral mortalities caused by these extreme thermal anomalies in the last 30 years [16,37,38,39,40,41,42]. Yet, some ETP reefs reveal fast recovery [9,12,16,41,43,44,45,46], and life history patterns of certain branching and massive coral reef species have shown high thermal tolerance thresholds [14,16,47,48,49,50]. Hence, assisted coral restoration with more resistant coral genotypes can be a pantropical conservation priority, to accelerate natural recovery and, also, to restore already-degraded reefs for improved future statuses of the ETP coral reef communities.

The number of active restoration projects in the ETP has increased dramatically in the last decade [51,52,53,54,55,56,57]. Yet, more studies are required in order to integrate and enhance restoration efforts in the region and other remote and marginal reefs, where accessibility to absent or scarce infrastructures and facilities is limited, and active restoration measures are not commonly implemented [58,59]. In the present study, we tested the efficiency of the direct outplanting of coral micro-fragments taken from *Pavona clavus* (massive, slow-growing species), *Pocillopora* cf. *eydouxi*, and *Pocillopora* cf. *effusus* (branching, fast-growing species), as a practical approach to restore remote and marginal ETP coral reefs. Annual increment of coral growth parameters as height (cm), width (cm), live tissue cover (cm^−2^), annual extension (cm year^−1^), skeletal density (g cm^−3^), annual calcification rate (g cm^−2^ year^−1^), survival, and self-attachment rates (%) were assessed over 13 months (400 days) of restoration (2018–2019) at the Islas Marías archipelago, Mexico, in the Northeastern Tropical Pacific.

## 2. Materials and Methods

### 2.1. Study Area

**Figure 1:**
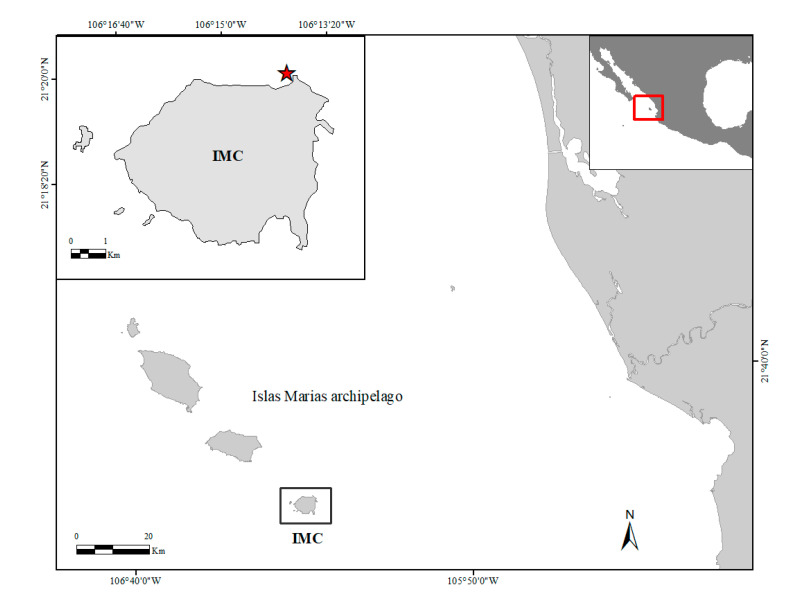
Restoration site (red star) located at Islas María Cleofas (IMC), Islas Marías archipelago in the Central Mexican Pacific (red box).

The study was conducted from June 2018 to August 2019 (400 days) at Islas María Cleofas (IMC), Islas Marías Biosphere Reserve in the Central Mexican Pacific, located 132 km offshore to the nearest coast of Nayarit, Mexico (Figure 1). The Marías archipelago is a restricted zone, being a federal prison area for over 100 years and where conservation measures on the marine ecosystems have been extreme [60]. This insular zone acts as a hub for coral species, and as a stepping-stone for larval dispersal route across the Central to Eastern Pacific regions and between Eastern Pacific coral communities [16,61,62]. Within this area, Islas María Cleofas harbors a high diversity of scleractinians, including branching *Pocillopora* species, in shallow waters (2–6 m), and massive *Pavona* and *Porites* species on deeper reefs [61,63]. The Marías archipelago is located in an ocean convergence zone influenced by interannual transitional ocean currents, the California current with seawater temperature (SWT) ranging from 18 to 21°C during December–April [64], by the Mexican coastal current with warmer SWT (~27–31 °C) between July–November [65], the seasonal upwelling during April–May [66] in addition to recurrent heat waves associated with intense ENSO (both “El Niño and La Niña” phases [67]), and by a high frequency of tropical cyclones [68].

### 2.2. Coral Fragment Transplantation

**Figure 2:**
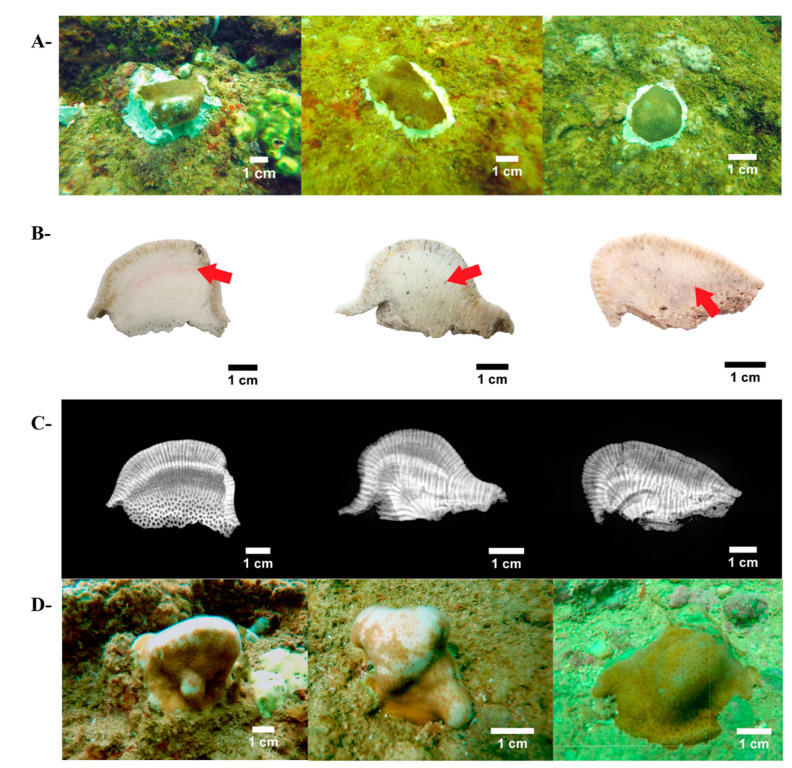
The use of micro-fragments of *Pavona clavus* from Islas Marías Cleofas for coral reef restoration. (**A**): micro-fragments at onset, just glued to the substrates; (**B**): coral skeleton slabs exhibiting Alizarin red marks (red arrows); (**C**): X-ray images of coral skeletons displaying annual bands pattern (light/dark shade = low/high density): (**D**): final coral size of the same colonies as in part A, 13 months later.

**Figure 3:**
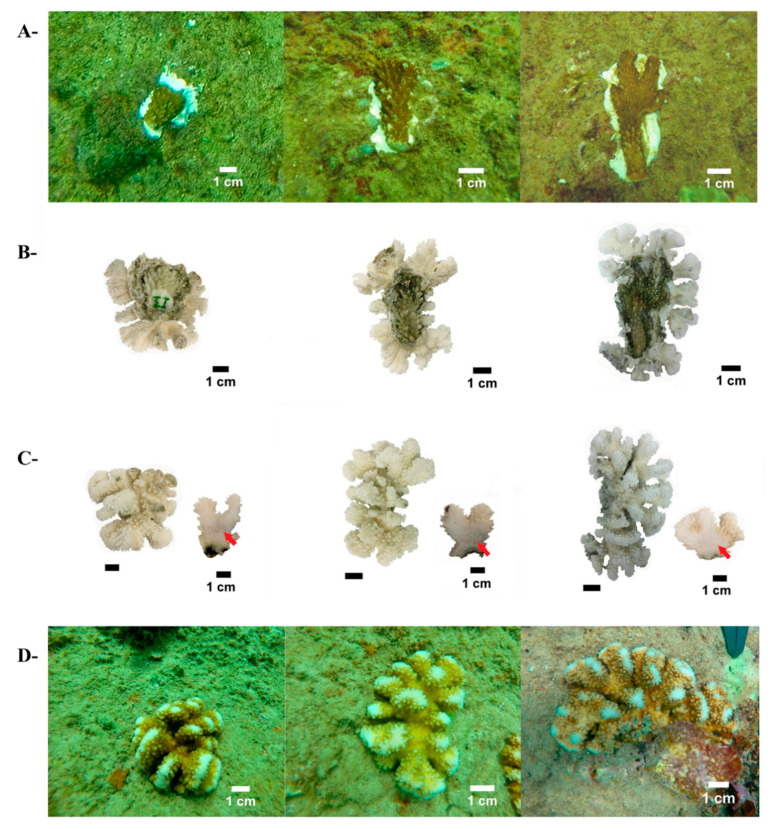
The use of micro-fragments of *Pocillopora* spp. from Islas Marías Cleofas for coral reef restoration. (**A**): micro-fragments at onset, just glued to the substrates; (**B**): coral skeletons comparing initial and final branches (bottom-side view); (**C**): coral skeleton upside view and coral branch slabs exhibiting Alizarin red marks (red arrows); and (**D**): final coral size of the same colonies as in part A, 13 months later.

Transplantation of coral species was performed with coral fragments taken from the main reef-building corals residing in Islas Marías, the massive species *Pavona clavus* and the branching species *Pocillopora* spp. Small portions (~3–5, less than 5% of *Pavona* colonies) of apical nodules and laminar edges (fragments ~25–30 cm^2^ each) were removed from 15 adult *P. clavus* colonies, using hammer and chisel. For the branching species, we collected naturally dispersed “corals of opportunity” fragments (~10–15 cm length) from 25 colonies of the morphospecies *Pocillopora* cf. *effusus* and *Pocillopora* cf. *eydouxi*. Collected fragments were removed and stained for 1 h ex situ (on board) with Alizarin Red (20 mg L^−1^, sigma) in aerated aquariums filled with local seawater, and translocated to the reef, where they were further fragmented to small coral tissue segments of sizes >3 cm^−2^ (coral micro-fragments), using a small, sharp chisel, following the recommendation by Forsman et al. [69]. We obtained 78 micro-fragments from *P. clavus* with a mean size of 4.5 cm^2^, and 3.2 cm^2^ for *Pocillopora* micro-fragments (*n* = 76) that were randomly arrayed along two plots at 5 m depth of ~6 m^−2^ each, with distances of ~5–10 cm between fragments. The fragments were glued to the natural substrate (limestone rock), previously cleaned manually by brushes (Figure 2A and Figure 3A), using a water-resistant silicon adhesive (MS Express, Fischer).

### 2.3. Coral Growth Metrics

Each of the 154 micro-fragments were tagged, and information was gathered on growth, survival, and attachment efficiency at three time points (at day 0, 32, 252, and 400). For coral growth, maximum height increase (h) was determined by the longest apical distance (cm) from base to the top of the colony, and maximum width (w) referred as the perpendicular length distance (cm) to the height axis, measured in situ with calipers (precision: 0.05 mm). Increment of live tissue surfaces (cm^−2^) was calculated using mean “h” and “w” values for all corals. The number of new adjacent branches in *Pocillopora* was also noted. Coral survival (%) was determined as the percentage of fragments with continuous live tissue growth along the experiment. Seawater temperature (SWT) was registered in situ daily, using underwater temperature loggers (HOBO) installed at the study location.

### 2.4. Calcification Rates and Annual Banding Measurements

After 13 months under natural conditions, 28 developing coral colonies (*Pavona clavus* (*n* = 15) and *Pocillopora* spp. (*n* = 13)) were collected (August 2019) and transported to the laboratory, their tissues were removed with fresh water jets, and skeletons were dried using pressurized air. Dried skeletons were then placed in a conventional oven at 75 °C for 3 h, in order to eliminate organic residues.

To determine annual banding and skeletal parameters for massive corals, each colony was cut into slices of 8–10 mm width, using a tipped-diamond saw blade (Qep) with fresh water as lubricant. Coral slices were X-rayed using a Philips X-ray machine (Mobile Diagnost Opta), set at 50 kv for 20 mAs at 1.8 m distance from the X-ray source (Figure 2C). In each X-ray, a wedge of the bivalve *Tridacna maxima* was used as a bulk density standard (2.77 g cm^−3^). X-ray images (75 dpi) were corrected using Duprey et al.’s [70] protocol, in order to eliminate irradiation bias (heel effect and square law) that may affect calculating density data. Afterward, the corrected images were analyzed, and skeletal density values were obtained (g CaCO_3_ cm^−3^) using the software ImageJ ver. 1.52s (National Institute of Mental Health, Bethesda, Maryland, MD, USA) (https://imagej.nih.gov/ij/), following Carricart-Ganivet and Barnes [71]. For annual extension rates (cm year^−1^), vertical distances were measured from the Alizarin red mark to the uppermost edge of each colonial skeleton with a digital caliper (Truper 0.001mm precision; Figure 2B). Annual coral calcification rates (g CaCO_3_ cm^−2^ year^−1^) were calculated by the product of mean annual extension rate and skeletal density [72].

Annual growth parameters for *Pocillopora* spp. were obtained from a subset of colony branches, which were cut below the Alizarin red marks and sliced using a handheld rotary cutting tool (Dremel 4000). Coral slices were photographed with a Canon Powershot XS500 camera (Canon Inc., New York, NY, USA), and the images were analyzed with ImageJ. Extension rates were determined by measuring the distances from the stain mark lines to the uppermost axes, in 3–6 branches for each colony (Figure 3C). Skeletal densities were measured in the same subset of branches using the buoyant weight method [73], and estimated as the mean dry weight (mass) divided by the differences between wet weight (water displaced) and dry weight. Coral calcification rates were calculated as for massive colonies, using the mean extension and density data set for each colony.

### 2.5. Data Analysis 

Mean average values of coral parameters (± standard deviation) were calculated, and all data were tested for normality (Shapiro–Wilk; *p* < 0.005) and homoscedasticity (Levene; *p* < 0.05). As data were not distributed normally nor homogenously, the non-parametric Kaplan–Meier function [74] was used to compare survival between branching and massive corals. Non-parametric ANOVA based on ranks (Kruskal–Wallis) was used to assess difference of coral growth metrics (density, extension, and calcification) among species. Simple linear regression tests were used to determine relationships between coral parameters, and the relation of coral growth with SWT. The constant variance and normality of residuals were evaluated for each regression. Statistical analyses were conducted using Sigma Plot Ver. 11 software (Systat Software, Inc., Chicago, IL, USA), with a confidence interval of 95% (α = 0.005). 

## 3. Results

**Figure 4:**
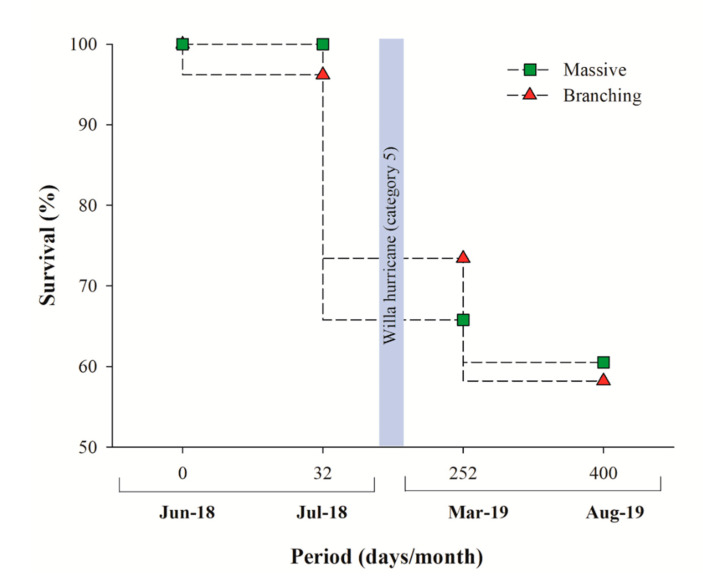
Coral survival (%) for *Pavona clavus* (green squares) and *Pocillopora* species (red triangles) during the 13-month in situ period. The vertical grey bar depicts Hurricane Willa date (118 restoration days).

A total of 154 coral micro-fragments were produced and outplanted within 3 days (30 h teamwork of two people), with five micro-fragments outplanted per hour/person. The total cost of the whole restoration process (installation, materials, and monitoring cost) was US$605 (~US$4.00 per outplanted coral, excluding indirect expenses (travel, accommodation, food supplies, and scuba gear costs). Pooled survivorship after 13 months was 60% (Figure 4), with no significant difference between *Pocillopora* spp. (58%) and *Pavona* (61%) corals (log rank, *X*^2^ = 0.333; *p* = 0.564). An increase in mortality (from 98% survivorship to 70% for *Pavona* and *Pocillopora* species) was observed following Hurricane Willa [75], which passed over the Islas Marías archipelago in October 2018, including the restoration site (Figure 4). Even though the accumulated mortality was modest, most (30%) dead fragments were dislodged, most probably by the storm, and were lost. Only 10% of micro-fragments died during the 13-month study period, following which they were fouled by macroalgae.

**Figure 5:**
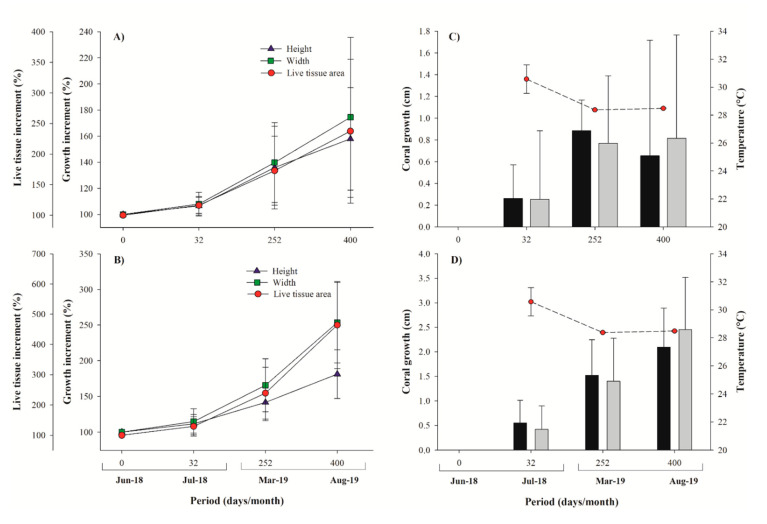
Micro-fragments growth measurements. Percentage of growth increment (% ± SD) of *Pavona clavus* (**A**) and *Pocillopora* spp. (**B**). Mean coral growth (cm ± SD) and seawater temperature at each interval time (days) for *Pavona clavus* (**C**) and *Pocillopora* spp. (**D**).

The *Pocillopora* growth measurements revealed a 183% linear extension and a 253% width increase, compared to initial sizes, and with average extension highs of 4.16 ± 1.02 cm (eight new branches developed from a single branch, one-year growth values; Figure 5, Table 1), 4.25 ± 1.39 cm for widths, and surface area of 5.27 ± 1.96 cm^−2^ (increased by 464%). *Pavona clavus* hemispherical structures increased by 158% and 174% in height and width, respectively. It presented an average growth rate of 1.66 ± 1.22 cm for height, and 1.65 ± 1.40 cm for width, a mean accumulated surface area of 5.27 ± 1.96 cm^−2^, and live coral tissue increase of 237%, with respect to initial measurements (Figure 5, Table 1). We further documented that live coral tissues completely overgrew the silicon-adhesive lugs by day 32, primarily in *Pocillopora* fragments and almost all (96%) of the micro-fragments (*Pocillopora* and *Pavona*) were self-attached to natural substrata within 3.9 months (range 1–8 months; Figure 5, Table 1). 

**Figure 6:**
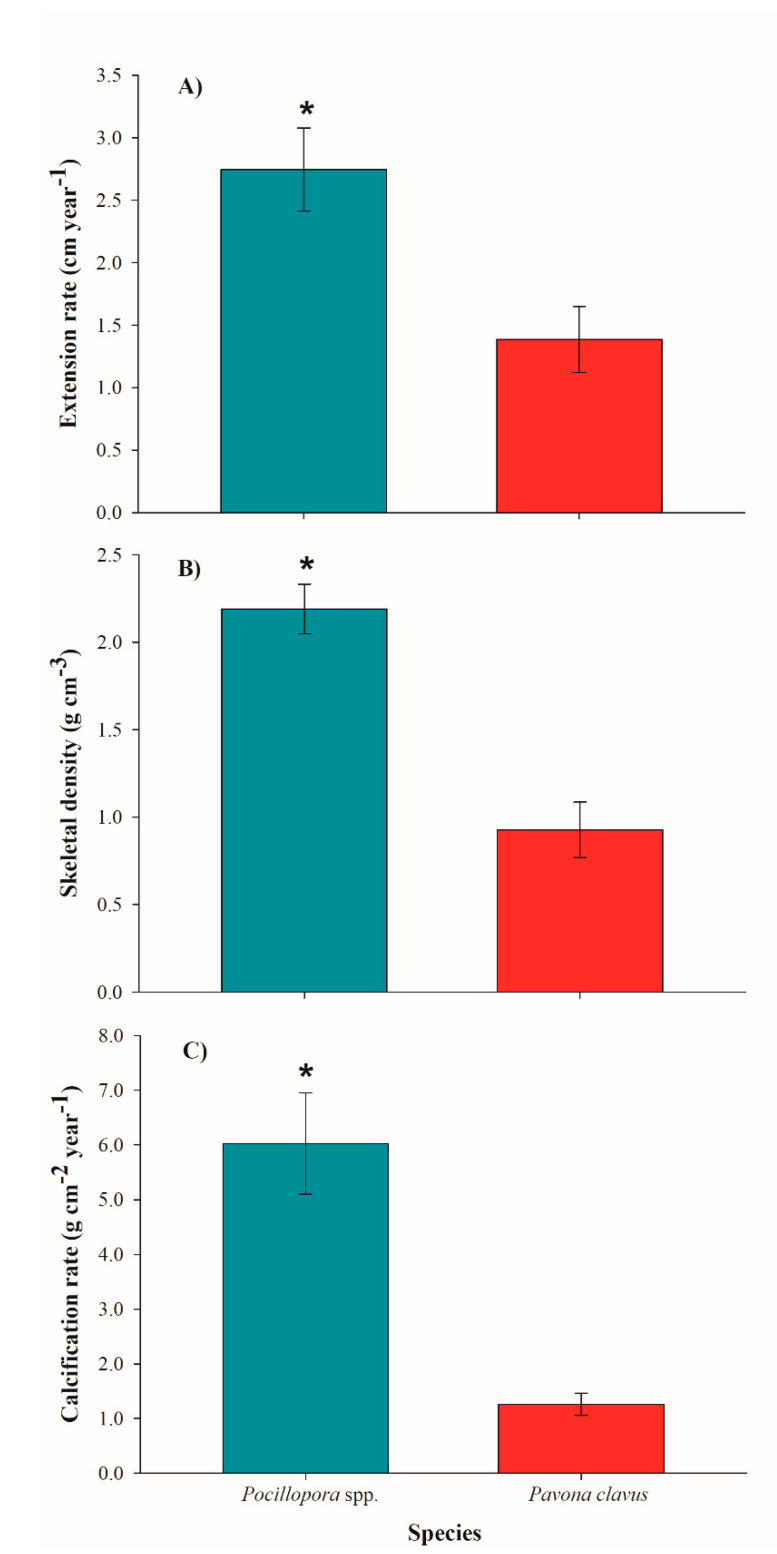
Annual growth parameters (±SD) for *Pocillopora* spp. and *Pavona clavus*. (**A**): annual extension rates; (**B**): skeletal density; and (**C**): calcification rates. Asterisks (*) denote significance differences between species (*p* < 0.001).

Yearly mean extension rates for *Pocillopora* spp. were 2.74 ± 0.33 cm year^−1^, with skeletal density of 2.19 ± 0.14 g cm^−3^, and calcification rate of 6.02 ± 0.93 g cm^−2^ year^−1^, compared to mean extension rates of 0.92 ± 0.16 cm year^−1^, skeletal density of 1.39 ± 0.25 g cm^−3^, and calcification rate of 1.25 ± 0.21 g cm^−2^ year^−1^ for *P. clavus* (Table 1). As expected, *P. clavus* revealed significantly lower growth rates, as compared to *Pocillopora* species in all three parameters: extension (H = 80.568: *p* < 0.001), skeletal density (H = 82.211: *p* < 0.001), and calcification rate (H = 81.397: *p* < 0.001) (Figure 6). For the *Pocillopora* species, we found positive correlations between extension and calcification rate (R^2^ = 0.82: *p* < 0.001) and density vs. calcification (R^2^ = 0.42: *p* < 0.001), but not for extension vs. density (R^2^ = 0.07: *p* = 0.005). In *P. clavus*, the calcification rates were positively correlated with extension rates (R^2^ = 0.24: *p* = 0.002) and skeletal densities (R^2^ = 0.20: *p* = 0.006), yet extension rates and skeletal densities were negatively correlated (R^2^ = 0.29: *p* < 0.001). Mean annual seawater temperature was 28.71 ± 2.38 °C, with a minimum of 25.03 °C during winter season and a maximum of 31.67 °C during summer. The SWT data was positively correlated with coral growth values of *Pocillopora* spp. (R^2^ = 0.49: *p* < 0.001) and with live tissue values of *P. clavus* (R^2^ = 0.12, *p* = 0.013).

## 4. Discussion

Global climate change and local-scale anthropogenic stressors have caused the unprecedented decline in coral reefs in the last three decades [5,7]. As natural recovery is slow, the emerging cost- and time-effective ecological restoration tools are of prime importance to mitigate coral reef degradation, and to boost reef biodiversity and ecosystem functionality recovery [15,23,25,33]. The literature further reveals two general attitudes that are employed: the simpler and cheapest way of direct transplantation of corals fragments to degraded reef [36,76,77] and the “coral gardening” approach that requires additional work and extended periods for coral transplantation through an intermediate aquaculture nursery phase, where coral fragments are cultured until reaching suitable transplantation sizes [18,27,78,79]. Many of the widely-used and efficient restoration techniques, such as the coral gardening and farming tenet [17,22,27,33,35,78,80], are time-consuming, and need constant maintenance of farmed corals and accessibility to infrastructure and facilities. This is impractical in cases, where, for example, restoration activities are performed in remote reefs or under unfavorable conditions, further highlighting the need to expand the restoration toolbox and to adapt alternative approaches, such as direct transplantation. 

The direct transplantation approach, while bypassing the need for established infrastructure and the creation of stock material for transplantation, has been criticized for results inconsistency, in terms of efficiency and effectiveness [81,82]. The production of micro-fragments (either for massive or branching corals) is commonly manifested in costly land-based coral farms (including cost for tanks, electricity, technicians, water-pumps, filters, and infrastructure maintenance) along extended periods that, with transportation and outplanting tools, may sum to US$13.00–61.00 per coral outplanted ([23], but also see [28]). Yet, there is no evidence that land-based coral farms enhance survivorship and growth rates after outplanting [36,76,77]. In contrast, the in situ production and direct transplantation approach, as presented in this study, is cheaper and more practical in terms of time (in the present work, US$4.00 per coral and 5 transplants per h per person), bypassing the use of nursery phase or the addition of artificial materials and substratum stabilization [17,23,25]. This is a real time-saving, effective and low-cost approach, with scalable options, preferable for remote reef. 

The overall first 13 months survivorship (60%) is similar to results obtained in previous, more time-consuming restoration approaches [17,25,30,51,52,53,56,83,84,85], even though the actual survival is much higher (about 90%), as most coral mortality was associated with fragment dislodgements (30%) due to the Willa hurricane forces, reflecting the impacts of natural catastrophes on other restoration projects [29,86]. The 10% of natural coral mortality is most probably due to competition with macroalgae (*Padina* sp.) overgrowth at the end of the restoration period (Figure 4). Thus, this study’s results further reveal a higher survival (20–40%), as compared to one year of outplanting in studies that use coral micro-fragmentation [36,76,77].

After one-year in situ, *Pocillopora* transplants showed a twofold increase in coral sizes (height and length), and *Pavona clavus* colonies almost doubled in width and length, and augmented 2.5-times in live tissue cover (Figure 2 and Figure 3). Notwithstanding, while the branching species grew three times faster than the massive species, the growth rates of all coral species used here revealed similar growth rates to previous successful restoration programs in the ETP region [52,53,57,87,88]. This fact is consistent with the tenet that micro-fragments and coral nubbins exhibit faster growth rates, compared to large fragments or whole colonies, a trait developed to avoid competition impacts or to reduce partial predation sways [69,77,89,90,91]. This trait further facilitates coral nubbins self-attachment on all types of substrata [92,93].

In the same way, annual extension (2.74 cm year^−1^) and calcification rates (6.03 g cm^−2^ year^−1^) obtained for *Pocillopora* spp. are similar to other documented outcomes in the ETP region [14,56,87,93,94,95]. The same applies to *Pavona*, one of the fastest growing massive coral species in the ETP region [49,87,88,94,95,96,97]. The consistency in the comparisons is further applied to coral density for branching (1.90–2.65 g cm^−3^) and massive (0.97–1.95 g cm^−3^) species [14,49,88,96]. Our results thus reveal the possible equal calcification patterns for both extension rates and skeletal density in coral nubbins, a different calcification landscape when compared to adult colonies, where calcification priorities are diverted between extension rates vs. skeletal densities [14,72,98].

## 5. Conclusions

Active coral reef restoration is becoming a key strategy to rehabilitate anthropogenic and climate change impacts [15,19,28,33,35]. Direct outplanting of micro-fragments and coral nubbins from keystone species, as studied here at the Islas Marías archipelago, has emerged as an effective, suitable, and affordable coral reef restoration methodology, primarily (a) in remote and marginal reefs less accessible to activities and methodologies performed in easily-reached reefs, and (b) when corals of opportunity are frequently used. Yet, the use of direct outplanting of coral nubbins and micro-fragments is still in its initial stages, and more comprehensive studies in additional sites worldwide are required in order to improve and to establish this new restoration avenue [36,76,77]. Direct transplantation of coral fragments in locations where time and conditions are limited, aiming to rehabilitate and support self-sustainable reef processes without available infrastructure, is thus an alternative practical tool for reef restoration in remote and marginal coral reef communities, such as the Islas Marías archipelago.

## Figures and Tables

**Table 1:** Growth parameters (±SD). Height (H), width (W), live tissue (LT), and attachment rates (AR) for *Pocillopora* spp. and *Pavona clavus*, along three time points.

Species	Day 32				Day 252				Day 400			
	H (cm)	W (cm)	LT (cm^−2^)	AR (%)	H (cm)	W (cm)	LT (cm^−2^)	AR (%)	H (cm)	W (cm)	LT (cm^−2^)	AR (%)
*Pocillopora* spp.	0.55 ± 0.46	0.42 ± 0.48	0.27 ± 0.59	10	2.07 ± 0.86	1.82 ± 0.99	2.53 ± 3.02	99	4.16 ± 1.02	4.25 ± 1.39	5.55 ± 3.34	100
*Pavona clavus*	0.21 ± 0.30	0.19 ± 0.28	0.13 ± 0.24	0	1.20 ± 0.94	1.02 ± 0.60	1.57 ± 2.13	94	1.69 ± 1.09	1.77 ± 1.23	3.59 ± 5.14	100
